# A Case of Right Superior Lumbar Hernia in an Elderly Woman: Differentiation from Lipoma in an Outpatient Setting with Bedside Ultrasonography

**DOI:** 10.31662/jmaj.2025-0251

**Published:** 2025-12-05

**Authors:** Masamichi Yoshika

**Affiliations:** 1 Yoshika Clinic, Osaka, Japan

**Keywords:** lumbar hernia, Grynfeltt’s hernia, elderly patient, lipoma differentiation, ultrasonography, internal medicine

## Abstract

Superior lumbar hernias (Grynfeltt’s hernias) are a rare type of abdominal wall hernia that may be under-recognized, particularly, by non-surgical physicians, because of their subtle and non-specific presentation. We report a case of an 82-year-old woman with a right-sided superior lumbar hernia initially suspected as a lipoma. The patient presented with a soft, painless mass on her right upper back that varied with posture, becoming prominent when standing and disappearing when supine. Physical examination showed a soft, mobile, non-tender subcutaneous mass. Although initially thought to be a lipoma, bedside ultrasonography revealed retroperitoneal fat protruding through a fascial defect without bowel involvement. Computed tomography confirmed the diagnosis of a right superior lumbar hernia. Because there were no symptoms or signs of incarceration, conservative management was chosen. The lesion remained stable over an 18-month follow-up. This case emphasizes key differentiating features―such as postural variation and fascial defects―that can help distinguish lumbar hernia from lipoma, especially in outpatient internal medicine settings. Most reported lumbar hernia cases rely on computed tomography, which is the gold standard for diagnosis, but this case demonstrates that bedside ultrasonography can also be highly useful for early recognition. Given that non-surgeons often encounter subcutaneous masses in elderly patients, awareness of lumbar hernia as a differential diagnosis is critical. This case illustrates that careful physical examination, combined with bedside imaging, can lead to accurate diagnosis, even in non-surgical settings. Highlighting these distinguishing features can help non-surgical physicians avoid misdiagnosis of this rare but clinically relevant condition and improve early detection in general medical practice.

## Introduction

Lumbar hernias are rare abdominal wall hernias, accounting for less than 2.0% of all abdominal hernias ^(1), (2)^. Among these, superior lumbar hernias (Grynfeltt’s hernias) are the most common subtype. Their anatomical location and subtle presentation often lead to under-recognition, especially in general internal medicine settings

Subcutaneous masses are frequently encountered in elderly patients. Although lipomas are common, alternative diagnoses should be considered when the mass changes with posture. Hernias involving retroperitoneal fat may mimic lipomas but require different management ^(3), (4), (5)^.

We report a case of a right-sided superior lumbar hernia initially suspected as a lipoma. This case highlights the importance of considering lumbar hernia in differential diagnoses of back masses and shows the value of bedside ultrasonography combined with imaging for accurate diagnosis in outpatient internal medicine practice.

## Case Report

An 82-year-old woman with rheumatoid arthritis and hypertension presented with a soft lump on her right back, which was first noticed in the previous four months. The lump was asymptomatic, more prominent when standing, and disappeared when lying down.

**Figure 1.**
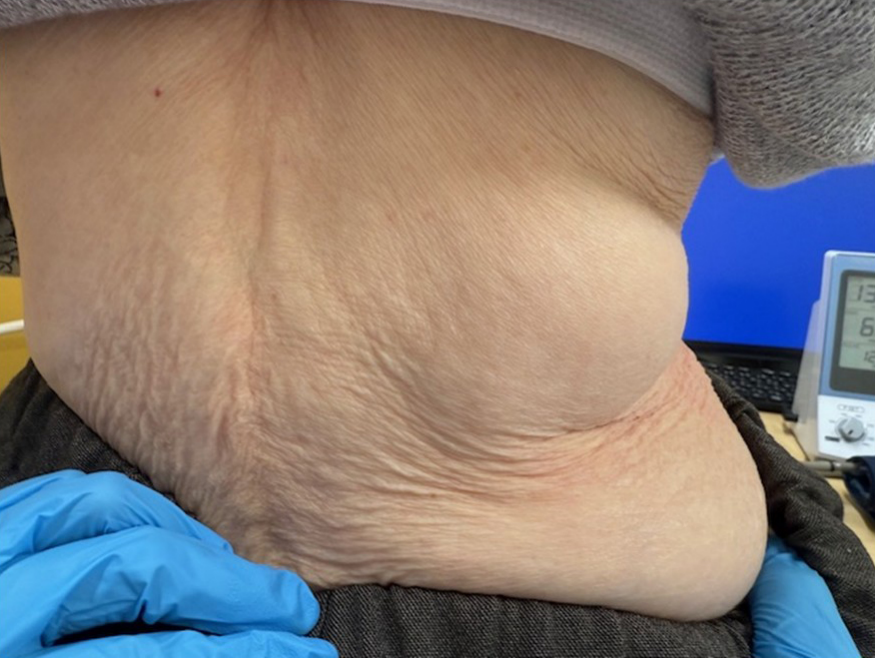
Clinical appearance of the right lumbar mass. A soft, protruding mass is observed in the right upper lumbar region while the patient is in a sitting position.

Physical examination revealed a soft, non-tender, mobile 4-cm mass in the right upper lumbar region, with normal overlying skin (Figure 1).

**Figure 2.**
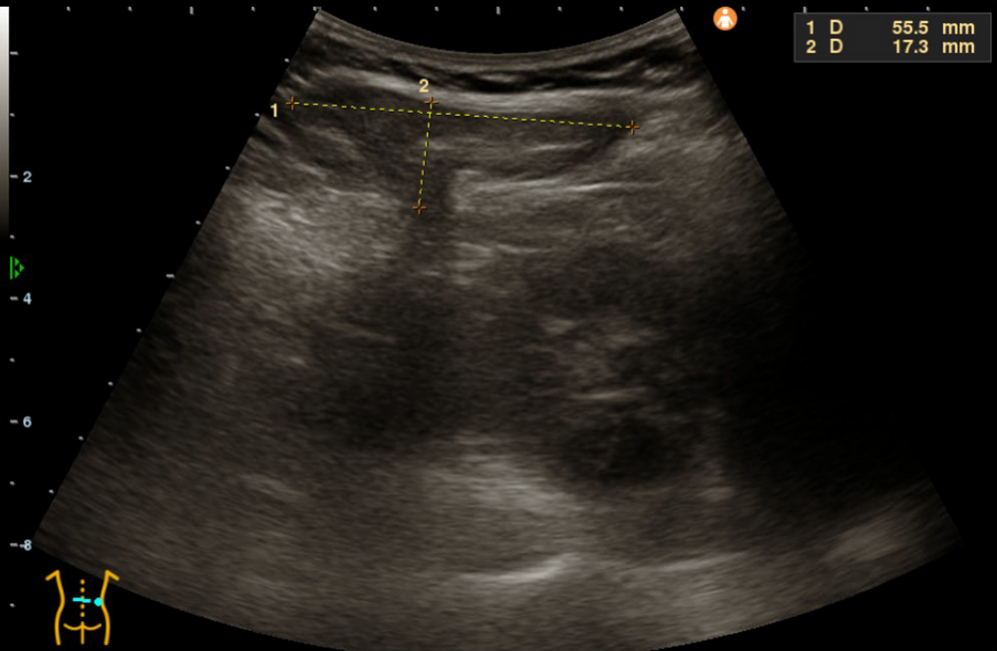
Ultrasonographic image showing a hyperechoic mass (measuring 55.5 mm × 17.3 mm) protruding through a fascial defect in the right superior lumbar region, consistent with herniated fat.

**Figure 3.**
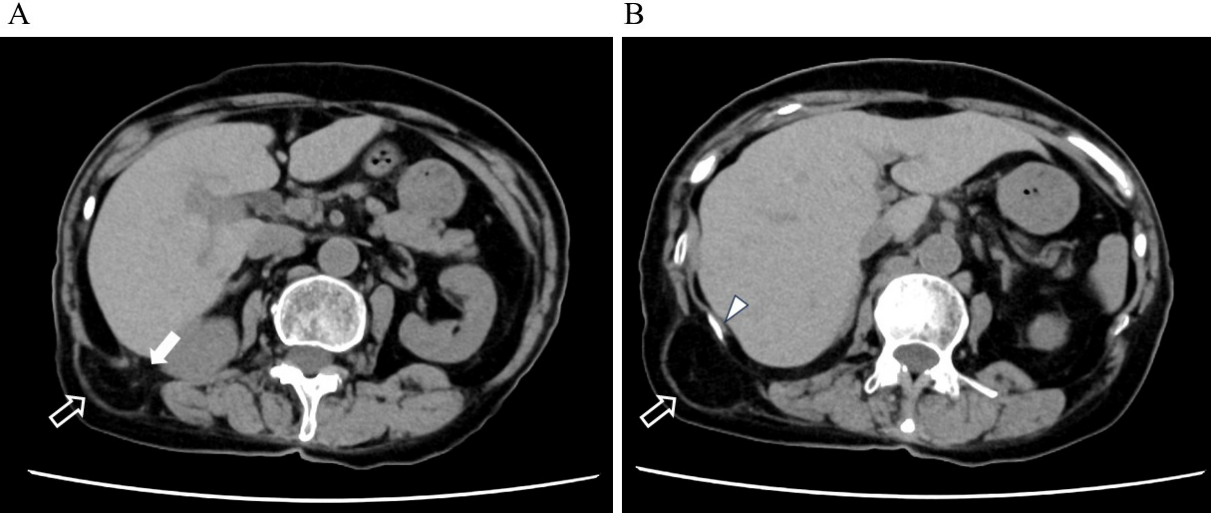
Axial CT images demonstrating a right superior lumbar hernia. (A) Retroperitoneal fat (hollow white arrow) is seen protruding through the hernial orifice (solid white arrow), located posterior to the right kidney and lateral to the liver. The fat extends into the subcutaneous tissue through the presumed location of the superior lumbar triangle (Grynfeltt’s triangle). (B) A more cranial slice than (A), with the arrow indicating the 12th rib. The continuity of the fat protrusion in an upward direction is clearly observed, with no involvement of bowel or other intra-abdominal organs. CT: computed tomography.

**Figure 4.**
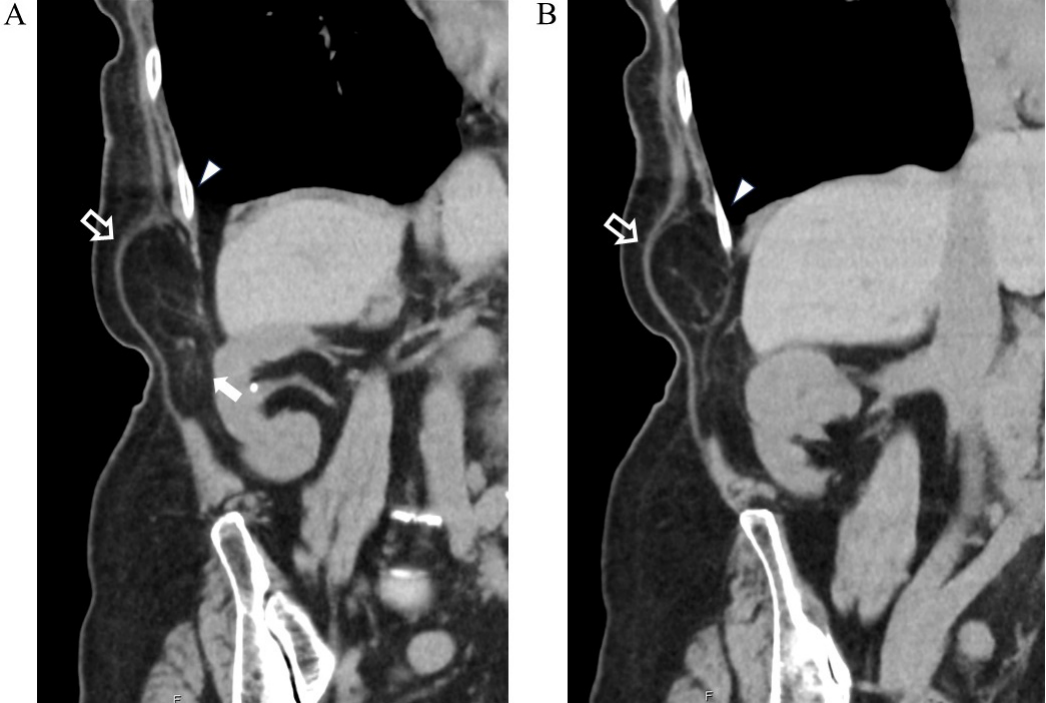
Coronal CT images of the same hernia. (A) The hernial orifice (solid white arrow) and protruding retroperitoneal fat (hollow white arrow) are shown. The fat extends continuously from the retroperitoneal space toward the subcutaneous layer. The 12th rib is also indicated. (B) A more posterior slice than (A), showing the retroperitoneal fat extending upward from the hernial orifice. CT: computed tomography.

Initially suspected as a lipoma, bedside ultrasonography revealed fatty tissue protruding through a 2-cm defect in the fascial layer without bowel involvement, consistent with lumbar hernia (Figure 2). Non-contrast computed tomography (CT) confirmed retroperitoneal fat herniation through the superior lumbar triangle between the quadratus lumborum and latissimus dorsi muscles (Figure 3). The hernial orifice was approximately 2.5 cm (Figure 4). A diagnosis of right superior lumbar hernia was made. Without bowel involvement or incarceration signs, conservative management was chosen. After 18 months, the hernia remained stable, with ongoing outpatient monitoring.

## Discussion

Superior lumbar hernia or Grynfeltt’s hernia is a rare hernia through the superior lumbar triangle bordered by the 12th rib, internal oblique, and quadratus lumborum muscles, with transversalis fascia forming the floor ^(1), (2)^.

Table 1 summarizes the characteristics of previously reported cases, including the present one ^(2), (3), (4), (5), (6), (7), (8), (9)^. Most superior lumbar hernias contained fatty tissue, although some included colon involvement. Uchida et al. ^(8)^ analyzed 114 Japanese cases and reported similar findings.

**Table 1.** Summary of Idiopathic Lumbar Hernia Cases from Case Reports and Retrospective Series.

Age	Sex		Hernia type		Side			Hernia contents	Reference
	Male	Female	Superior	Inferior	Right	Left	Bilateral		
87		1	1		1			Extraperitoneal fat	2
79		1		1	1			Extraperitoneal fat	3
79	1		1			1		Extraperitoneal fat	4
72		1	1			1		descending colon	5
31-81	6	10	Not listed		6	9	1	Extraperitoneal fat	6
5-79	13	15	25	3	11	15	2	Not listed	7
67		1	1		1			Extraperitoneal fat	8
47		1	1			1		Extraperitoneal fat	9
82		1	1		1			Extraperitoneal fat	This case

Individual case reports are shown with specific patient ages (e.g., 87, 79, 72), whereas retrospective case series are presented as age ranges (e.g., 31-81, 5-79). Sex distribution, hernia type, laterality, and contents are summarized accordingly. Reference numbers correspond to the cited literature.

Lumbar hernias account for less than 2% of all abdominal wall hernias, with approximately 70%-80% occurring in the superior lumbar triangle. They are most commonly observed in middle-aged to elderly individuals and show a slight female predominance. Left-sided hernias are reported more frequently, which is thought to be because of the limited mobility of retroperitoneal fat and viscera on the right side caused by the anatomical presence of the liver ^(2), (3), (6), (7), (8)^.

The present case involved an elderly woman, consistent with the typical demographic trends; however, the hernia occurred on the right side, which is considered less common. In addition to the hepatic constraint, previous studies have suggested that the muscle and fascial support structures on the right side of the body may generally be stronger than those on the left, thereby reducing the likelihood of herniation on the right ^(6)^. This enhanced structural resistance may contribute to the lateral asymmetry observed in reported cases.

This case highlights the anatomical variability of lumbar hernias and underscores the importance of imaging modalities in recognizing atypical presentations, particularly, in outpatient, non-surgical settings. In elderly individuals, age-related muscle atrophy, decreased elasticity of connective tissues, and increased intra-abdominal pressure because of chronic coughing or poor posture further exacerbate the vulnerability of the Grynfeltt-Lesshaft’s triangle. In addition, degeneration of the thoracolumbar fascia and surrounding muscle groups may promote the protrusion of retroperitoneal fat or intra-abdominal contents. In the present case, although there was no history of trauma or surgery, these age-related changes were considered to have contributed to the development of the hernia ^(7)^.

Differentiating lumbar hernia from lipoma is crucial. Both present as soft, painless, mobile masses; however, hernias may change size or shape with posture and often have a palpable fascial defect, unlike lipomas ^(3), (4), (5)^. These clinical clues led to further imaging.

Ultrasonography and CT were critical for diagnosis. Ultrasound, available at bedside in outpatient settings, identified fatty tissue protruding through the fascial defect, facilitating early recognition ^(9)^. However, most cases in literature rely on CT, which remains the gold standard for definitive diagnosis of lumbar hernias ^(1), (10)^. CT provides precise detail of hernia contents, defect size, and bowel involvement or incarceration.

No standardized treatment guidelines exist because of rarity. Surgery is indicated for symptomatic cases or bowel involvement. Early surgery is often advised to prevent complications; thus, literature mostly focuses on surgical management ^(2), (7)^.

Our patient had an asymptomatic right superior lumbar hernia composed only of fatty tissue without bowel involvement, located between the liver and kidney, with a low risk of incarceration. Conservative management was chosen per patient preference, with stable follow-up over 18 months.

Although rare, lumbar hernias should be considered in differential diagnosis of lumbar masses by internists and surgeons. This case demonstrates the clinical value of bedside ultrasonography for differentiating lumbar hernia from lipoma, a valuable tool accessible in outpatient general medicine. Early ultrasound recognition may enhance clinical diagnostic skills and image-based teaching.

We obtained written informed consent from the patient before manuscript preparation.

## Article Information

### Author Contributions

Masamichi Yoshika contributed to the study conception and design, performed data collection, made substantial contributions to data analyses and interpretation, and wrote this manuscript.

### Conflicts of Interest

None

### Approval by Institutional Review Board

Not applicable.

## References

[1] Sanders JV, Cavalcante JB, Lucena JD, et al. Clinical and surgical anatomy of lumbar hernia: a review. Int Arch Med. 2017;11:10.

[2] Piozzi GN, Cirelli R, Maino MEM, et al. Management criteria of Grynfeltt’s lumbar hernia: a case report and review of literature. Cureus. 2019;11(1):e3865. 30899616 10.7759/cureus.3865 PMC6414194

[3] Kadler B, Shetye A, Patten DK, et al. A primary inferior lumbar hernia misdiagnosed as a lipoma. Ann R Coll Surg Engl. 2019;101(4):e96-8. 30773901 10.1308/rcsann.2019.0009 PMC6432969

[4] Heo TG. Primary Grynfeltt’s hernia combined with intermuscular lipoma: a case report. Int J Surg Case Rep. 2021;84:106163. 34225060 10.1016/j.ijscr.2021.106163 PMC8261650

[5] Stupalkowska W, Powell-Brett SF, Krijgsman B. Grynfeltt-Lesshaft lumbar hernia: a rare cause of bowel obstruction misdiagnosed as a lipoma. J Surg Case Rep. 2017;2017(9):rjx173. 28928928 10.1093/jscr/rjx173 PMC5597899

[6] Chen ZM, Fan XQ, Zhou YX. Retrospective analysis of 16 cases of lumbar hernia. Heliyon. 2023;9(11):e22235. 38045220 10.1016/j.heliyon.2023.e22235 PMC10692800

[7] Shen C, Zhang G, Zhang S, et al. Clinical, surgical characteristics and long-term outcomes of lumbar hernia. BMC Surg. 2021;21(1):332. 34445979 10.1186/s12893-021-01328-7 PMC8394050

[8] Uchida T, Otsuka H, Tani S, et al. A case of idiopathic superior lumbar hernia. J Jpn Surg Assoc. 2007;68(10):2388-92. Japanese.

[9] Li J. The role of ultrasound in the diagnosis of Grynfeltt-Lesshaft lumbar hernia: a case report. Australas J Ultrasound Med. 2021;24(3):178-80. 34765428 10.1002/ajum.12276 PMC8409450

[10] Aguirre DA, Casola G, Sirlin C. Abdominal wall hernias: MDCT findings. AJR Am J Roentgenol. 2004;183(3):681-90. 15333356 10.2214/ajr.183.3.1830681

